# Efficient Multitask Onboard Vision Sensing for Open-Pit Mining Advanced Driver Assistance System with Classification-Guided Adaptive Temporal Inference

**DOI:** 10.3390/s26123860

**Published:** 2026-06-17

**Authors:** Maximiliano Vélez, Claudio Urrea

**Affiliations:** Electrical Engineering Department, Faculty of Engineering, University of Santiago of Chile, Las Sophoras 165, Estación Central, Santiago 9170124, Chile; maximiliano.velezm@usach.cl

**Keywords:** onboard vision sensors, vehicular sensing, ADAS, driver support system, multitask deep learning, drivable area segmentation, steering classification, video semantic segmentation, adaptive temporal inference, open-pit mining

## Abstract

Cameras and IMUs on heavy mining trucks supply the visual signal that Advanced Driver Assistance Systems (ADASs) use in open-pit operations. Haul roads in a surface mine are unstructured and unmarked, so a perception model must be both accurate and fast. We address this with a video-based multitask pipeline for a mining Driver Support System (DSS): a single BiSeNetV1 network produces drivable-area segmentation and steering-direction classification in one forward pass. Training used only 100 frames sampled non-sequentially from in-cab recordings of a real open-pit mine; evaluation used two full onboard sequences. To exploit temporal redundancy without annotating video, we propose an Adaptive Clockwork (A-CW) inference scheme: the spatial path runs on every frame, while the context path is refreshed only on keyframes whose cadence is set by the classification output, the same signal shown to the driver as a steering hint. This classification-guided policy increases context updates on curved segments, where the scene changes more rapidly, and reduces them on straight sections, where semantic redundancy is higher. The selected A-CW configuration was evaluated on full temporal test sequences, including one route kept entirely outside the training source. On this unseen route, A-CW achieved 94.70% road-class IoU and 73.68% Top-1 Accuracy. GPU-only throughput increased from about 55 FPS with frame-by-frame inference to 168.01 FPS, and display-excluded end-to-end processing in the simulated ADAS pipeline remained at approximately 37.5 FPS.

## 1. Introduction

The forward-facing camera of a haul truck is, in practical terms, the primary perception sensor of the vehicle. Together with an Inertial Measurement Unit (IMU) and the occasional auxiliary depth sensor, it generates the continuous data stream that an Advanced Driver Assistance System (ADAS) must interpret to be of use on unstructured haul roads with weak visual cues and almost no signage. The value of such a system depends, ultimately, on how quickly and reliably that onboard imagery can be converted into driving-relevant estimates. Open-pit mining has attracted growing attention from the sensing and intelligent-transport communities: examples range from camera-based extraction of mining-area boundaries [1] and deep-learning-based mapping of haul roads for operational planning [2] to autonomous driving fully integrated into the mining workflow, which has been reported to extend productive hours [3]. In this same direction, ADAS [4] sits as the natural intermediate step between manual driving and full vehicle autonomy [5]. These systems reduce the attentional load on the operator and help prevent the kinds of errors that lead to accidents [6], unscheduled stops, and operational losses—costs that, in mining, are sizeable [7].

Several complementary taxonomies have been proposed for ADAS [8]: by functional class (longitudinal/lateral control, monitoring, information systems), by the kind of interaction with the user (informing, warning, intervening, comfort-enhancing), or along continuous axes such as urgency and level of control. A widely used distinction at the system level separates Driver Monitoring Systems (DMSs) [9], which try to estimate the psychophysical state of the driver in order to mitigate inattention-related accidents, from Driver Support Systems (DSSs) [10,11], which intervene on the driving task itself: longitudinal control (acceleration and braking) and lateral control (guidance and steering) [12].

**Figure 1:**
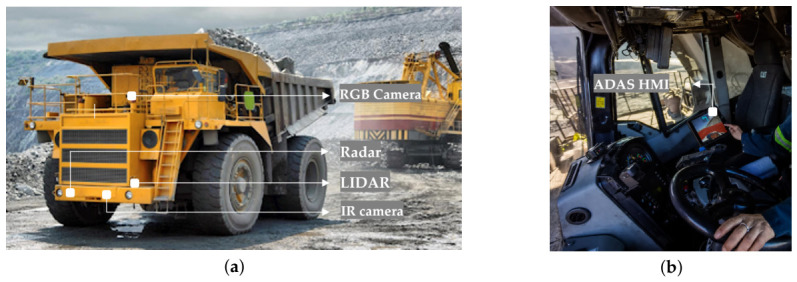
Equipment schematic for a DSS-type ADAS implementation on an open-pit mining dump truck: (**a**) Mining dump truck and base sensors for the DSS-type ADAS system implementation. (**b**) Section of the truck cabin where the driver interacts with the ADAS system through the HMI.

Figure 1 shows a DSS-type ADAS prototype deployed on a haul truck operated in an open-pit mine. The vehicle carries a set of exteroceptive sensors—RGB cameras, LiDAR [13], and, depending on the configuration, complementary radar or thermal imaging—whose outputs feed a perceptual representation of the surroundings into a Human–Machine Interface (HMI) [14]. Through that interface, the driver receives assistance cues: an estimate of the drivable area [15], a recommended steering direction [16], and obstacle tracking with preventive risk or collision alerts [17].

Between the sensors and the operator’s decision sits the perception stack, which turns raw data into estimates that are useful for driving. These usually combine semantic segmentation of the scene [18], object detection along the route [19], and the classification of conditions or events of interest for driving [20]. All of this has to run on the same vehicle, which is why multitask architectures that share representations across tasks have become the standard way to keep computational cost and latency under control [21].

Most image-based scene perception work, though, has been developed and validated on structured urban imagery. Onboard vision sensing in industrial environments such as open-pit mines has received much less attention, even when the sensing geometry, the statistics of the scene, and the operational constraints are quite different from those of urban driving. Mining scenarios demand robustness to variations of the visual domain: changes in road infrastructure, unmarked routes [22]—all without giving up real-time operation. Deep Learning (DL) has shown competitive performance on this class of problem by learning representations that adapt to the environment [23,24], and efficient designs already reach inference speeds compatible with real-time applications [25,26].

A sizeable fraction of these DL studies, however, still targets urban, large-scale, and relatively structured datasets, with experimental protocols that evaluate performance close to the training distribution [27,28,29]. Another limitation is that evaluation is, in most cases, frame-by-frame: the literature reports per-frame metrics, while the temporal behaviour of the system over sequences—and under realistic ADAS conditions, with the driver in the loop—is examined far less often [30].

The mining domain amplifies this gap. Unlike other driving contexts, there are no large, consolidated datasets that capture open-pit operation in enough detail and that, at the same time, provide extended sequences with annotations rich enough to train supervised temporal methods. Building such datasets is costly in acquisition, curation, and labelling time, which caps how far they can scale. This in turn motivates a closer look at inference strategies that exploit the temporal redundancy already present in video, without depending on densely annotated sequences at training time.

The present paper takes that as a starting point and studies a multitask perception model for a DSS-type ADAS for mining operations, with visual feedback through an HMI. The model is built around an efficient bilateral architecture and a temporal update strategy inspired by the Clockwork paradigm [31]. It combines a semantic-segmentation branch that estimates the drivable area with a classification branch that flags trajectory changes associated with scenes of higher perceptual variability. What departs from a conventional multitask formulation is the role of the classification output, which plays two parts at once: an assistance signal for the driver, conveying the expected steering direction over unmarked terrain, and an event-warning mechanism that adapts the model’s update frequency on the fly. When the system detects more demanding conditions—typically a curve—the deep features are refreshed more often; on quieter sections, they are refreshed less often and computation is saved. The selected adaptive strategy is also evaluated under focused conditions, covering video-segmentation baselines, hardware execution across GPU platforms, high semantic redundancy, and controlled visual degradations.

To the best of the authors’ knowledge, integrating semantic segmentation and steering-direction classification into a single multitask architecture that doubles as a driver-assistance output and as the control signal of an adaptive temporal update policy has barely been explored in autonomous-driving perception. The present work is the first to apply this idea to onboard image sensing in open-pit mining vehicles. The main contributions can be summarised as follows:

- A bilateral multitask Deep Learning (DL) architecture, based on BiSeNetV1, that integrates drivable-area semantic segmentation and steering-direction classification from in-cab camera imagery inside a single network, targeted at a DSS-type ADAS application in open-pit mining with visual feedback through an HMI.
- A classification-guided Adaptive Clockwork inference scheme for video-based perception. The scheme exploits inter-frame redundancy by running the spatial path on every frame and refreshing the context path only on keyframes. The classification head plays a double role: it provides the steering-direction cue for the ADAS interface and it sets the keyframe cadence according to the predicted trajectory condition. In practice, this policy shortens the update period on curved segments, where the scene geometry changes more rapidly, and lengthens it on straight sections, where semantic redundancy is higher. Because training does not rely on densely annotated sequences, the annotation requirements drop substantially.
- A compact, manually annotated dataset of onboard imagery from camera sequences recorded inside an open-pit mine, together with an evaluation protocol that uses isolated images for frame-by-frame validation and full onboard videos for temporal evaluation. The protocol is well suited to assessing efficient video-based perception under the limited-supervision regime typical of onboard vision sensing in heavy industry.

The remainder of this paper is organised as follows. Section 2 reviews related work on multitask learning, image classification within multitask schemes, and efficient video semantic segmentation. Section 3 presents the materials and methods: dataset, bilateral multitask architecture, Adaptive Clockwork inference strategy, training procedure, temporal baseline implementation, ADAS simulation protocol, and the focused evaluation setup. Section 4 reports the experimental results. Section 5 discusses what these results imply for onboard vision sensing in unstructured industrial environments. Section 6 closes with the conclusions, and Section 7 outlines future research directions.

## 2. Related Work

### 2.1. Multitask Learning Models for ADAS

**Figure 2:**
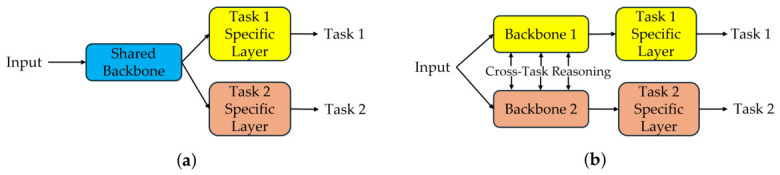
Types of architectures for implementing multitask learning models: (**a**) Architecture with hard parameter sharing approach. (**b**) Architecture with soft parameter sharing approach.

Multitask Learning (MTL) trains a single Deep Learning (DL) network on several related tasks simultaneously through a joint optimisation scheme [32]. At inference time, the network returns all predictions in a single forward pass, which cuts both computational cost and latency relative to running independent models. As sketched in Figure 2, MTL designs can either share a single backbone among tasks or assign a dedicated backbone to each task [33].

From there, recent research has moved towards designs intended for embedded deployment, with compact networks and inference times that fit real-time budgets. Gao et al. [34] combine YOLOv5 with DeepLabV3+ over a MobileNetV2 backbone [35] to lower computational complexity on unstructured roads. DriveLaneNet [36] follows a related path: it builds a lightweight model on MobileNetV3 [37] reinforced with an attention module, with a small parameter budget specifically targeted at resource-limited environments. The unified single-stage architecture in [38] fuses multiscale and inter-frame information through an FPN, sidestepping heavier alternatives. A complementary direction is taken in [39]: a real-time multitask scheme covers 3D detection, semantic segmentation, and depth estimation, and a Task-adaptive Attention Generator keeps negative transfer in check without pushing inference cost too high. The same line is reinforced in [40] via a YOLOv8 [41] backbone with a neck that combines ASPP and hybrid dilated convolution to improve feature fusion at high speed. YOLOP-DN [42] extends YOLOP [43] with SPPF modules [44] and a dual-neck design that keeps latency bounded; speed drops relative to the YOLOP baseline, but real-time operation is preserved, and the authors demonstrate deployment on an embedded device that reaches close to 18 FPS after FP16 quantisation.

Beyond the perception-focused designs above, recent work has also pushed MTL towards onboard visual streams of vehicles operating in non-urban scenarios. YOLOPv3 [45] proposes an efficient anchor-based network that jointly handles traffic object detection, drivable-area segmentation, and lane detection in a single forward pass, achieving state-of-the-art results on the BDD100K benchmark while preserving competitive inference speed. Even YOLOPv3, however, operates frame-by-frame, so inter-frame redundancy is left untouched and classification-guided temporal adaptation remains unexplored.

Across these works, the dominant choice remains frame-by-frame inference: every input is processed in isolation. This discards the temporal continuity of the visual stream, in which information from consecutive instants is strongly redundant and correlated [46].

### 2.2. Image Classification in Multitask Learning Models

Image classification assigns a single semantic label to an input image. It is one of the foundational tasks of computer vision, and many of its backbone feature extractors are re-used across complementary tasks such as detection and segmentation [47]. CNN-based and ViT-based backbones dominate the literature; whether a CNN or a transformer is preferable depends on the application and on the amount of training data available [48].

In the autonomous-driving and ADAS context, classification has been used as a stand-alone task to identify the road type [49], to recognise traffic signs [50], and to estimate the weather along the route [51]. Multitask schemes that pair detection with classification have also been reported, both for traffic-sign identification [52] and for perception under adverse weather [53]. Within the works we reviewed, however, none combine segmentation and classification in a single multitask model for this application.

Outside autonomous driving, segmentation and classification have been bundled into the same MTL network in other domains. In medical imaging, ref. [54] relies on a ResNet backbone and adds a contextual attention module to the decoder to sharpen the boundaries of diffuse lesions. For fault classification in microstructures, ref. [55] attaches two output heads to a U-Net-style architecture, with global pooling and a fully connected layer on the classification branch. Vinod et al. [56] apply both tasks to horticulture, with a ViT-based encoder and a segmentation decoder linked to the encoder by skip connections.

### 2.3. Efficient Video Semantic Segmentation Models

Image streams produced by vehicle-mounted cameras are a particular case of temporal sensor data: inter-frame redundancy can be exploited to cut the cost of full scene interpretation without losing accuracy. Most DL models for real-time Video Semantic Segmentation (VSS) still process each frame independently, which is computationally wasteful and ignores the strong correlation between consecutive frames [57]. Several feature-propagation strategies have been proposed to mitigate this, all of them based on the same idea: only some frames—the so-called keyframes—are fully processed to produce up-to-date deep features, while non-keyframes either re-use those features directly or receive them after propagation. Two main strategies dominate the literature: optical-flow-based propagation [58], which estimates motion to warp features from one frame to the next, and direct feature re-use [59], which exploits the temporal stability of part of the semantic information by preserving it across frames and updating only the components most sensitive to scene change.

These designs improve efficiency, but their performance depends critically on how keyframes are selected. Once the reference frame stops being representative of the current scene, propagated features lose validity and errors propagate. Recent work has therefore moved towards adaptive keyframe selection. In [60], a new keyframe is triggered when a lightweight network operating on the difference between low-level features predicts that the segmentation produced from the previous keyframe is no longer reliable. Gao et al. [61] use a Siamese network that scores the similarity between the current frame and the keyframe: when that similarity drops below a threshold, the keyframe is refreshed. An et al. [62] base the decision on the feature transfer capacity, measured via the Potential Prediction Error (PPE); a frame becomes a keyframe whenever propagation from the reference is no longer reliable enough. All three approaches need training on consecutive video sequences to learn both temporal propagation and the update criterion, which is hard to apply when only static images or non-sequential annotations are available.

## 3. Materials and Methods

### 3.1. Dataset Preparation

The imagery dataset was built from three scenarios of the AutoMine benchmark [63], a collection of onboard camera sequences recorded from a heavy mining truck operating in an open-pit environment. Each scenario contains synchronised RGB image streams and Inertial Measurement Unit (IMU) data, so both the visual sensing data and the kinematic state of the vehicle are available. The recordings are sampled at a temporal resolution of 0.1 s between consecutive frames.

From the three selected scenarios, we defined a training subset and an evaluation subset. For training, isolated images were sampled non-sequentially from two scenarios. For temporal evaluation, we kept two full sequences: one belonging to a scenario that had also contributed training images, and one belonging to a scenario kept entirely out of training. The complete traceability of scenarios, sequence IDs, and selected frames is provided in Appendix A.

To characterise the local geometry of the trajectory, the evaluation routes were divided into straight and curved segments based on the temporal evolution of the vehicle yaw angle. The yaw angle ($\psi_{i}$) at each instant $t_{i}$ was estimated from the orientation quaternion $q_{i}=\left(q_{x,i},\;q_{y,i},\;q_{z,i},\;q_{w,i}\right)$ delivered by the IMU, through the arctangent of Equation (1):

(1) $$\psi_{i}=\mathrm{atan}2\left(s_{i},\;c_{i}\right)$$

with

(2) $$s_{i}=2\left(q_{w,i}\;q_{z,i}+q_{x,i}\;q_{y,i}\right)$$

(3) $$c_{i}=1-2\left(q_{y,i}^{2}+q_{z,i}^{2}\right)$$

The rotation rate around the axis perpendicular to the vehicle, $w_{i}$, was then computed from $\psi_{i}$ as a discrete derivative via finite differences, Equation (4):

(4) $$w_{i}=\frac{\psi_{i}-\psi_{i-1}}{\Delta t_{i}};\;\Delta t_{i}=t_{i}-t_{i-1}$$

The training set was assembled with a non-sequential sampling criterion. Frames were taken every 8 s on straight segments and every 4 s on curved segments, so that turning events would be better represented. A total of 100 images were collected in this way.

**Figure 3:**
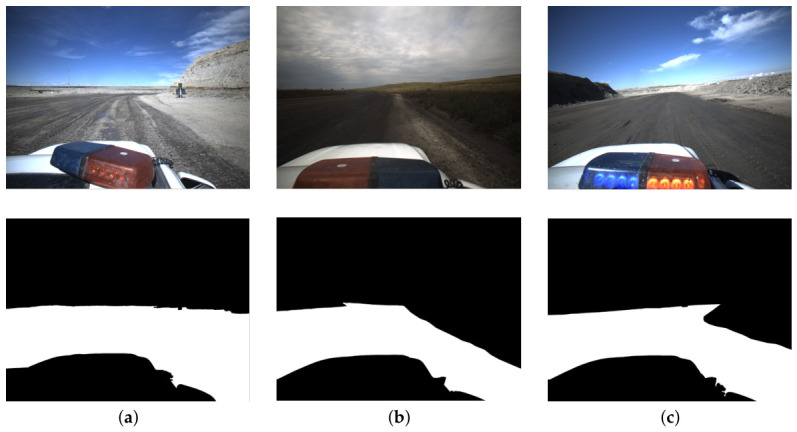
Examples of images and corresponding binary annotations from selected sequences in the mining environment: (**a**) Sample of sequence used for training and testing. (**b**) Sample of sequence used only for training. (**c**) Sample of sequence used only for test.

For the test sequences, by contrast, frames were annotated every 1 s, ensuring that the temporal evaluation of the method remains representative of its inference-time behaviour. As Figure 3 shows, the annotations are binary, distinguishing the drivable area from the rest of the environment. All masks were produced manually with the Label Studio tool [64].

Table 1 lists the sequences used for evaluation. We chose single-lane driving routes with no other vehicles or pedestrians; in this way, the most relevant scene changes during testing come from the trajectory geometry itself, i.e., from the transition between straight and curved segments. Route A lasts 103 s and contains two curves—one to the right and one to the left; a subset of its frames was also used for training, and it is therefore reported as a partially seen route. Route B lasts 56 s, contains a single left-hand curve, and was used exclusively for testing and is therefore reported as an unseen route. This distinction is indicated explicitly in Table 1 through the training-source overlap column.

This construction strategy—training on isolated frames, evaluating on full temporal sequences—fits the practical constraints of onboard vision sensing in heavy industry well: annotated sequential data is scarce and dense video labelling is expensive.

### 3.2. Adaptive Clockwork Model

This section presents the two components of the proposed model. The first is a bilateral multitask architecture based on BiSeNet V1 [65], designed to estimate the drivable area and the steering direction simultaneously. The second is the adaptive temporal scheme applied at inference time, which refreshes keyframes dynamically and re-uses contextual features across consecutive frames.

#### 3.2.1. Bilateral Multitask Architecture

The multitask architecture is built on BiSeNet V1, a bilateral network originally proposed for real-time semantic segmentation that combines computational efficiency with enough representational capacity for scene understanding. We picked BiSeNetV1 for two reasons: its favourable trade-off between perceptual quality and inference speed, and its track record on low-contrast scenes [66], where the boundary between the drivable area and the rest of the environment is barely visible. For the kind of onboard sensing the present application requires, BiSeNetV1 is a good fit. It keeps high-resolution spatial detail while still capturing the global semantic context, and that balance becomes important when the sensor itself is moving and the scene geometry shifts continuously along the vehicle’s path.

The network has two main branches: a context path and a spatial path. The context path captures high-level semantic information and widens the effective receptive field, allowing the global structure of the scene to be modelled. The spatial path preserves fine detail and high resolution—precisely what is needed to delimit the drivable area accurately.

**Figure 4:**
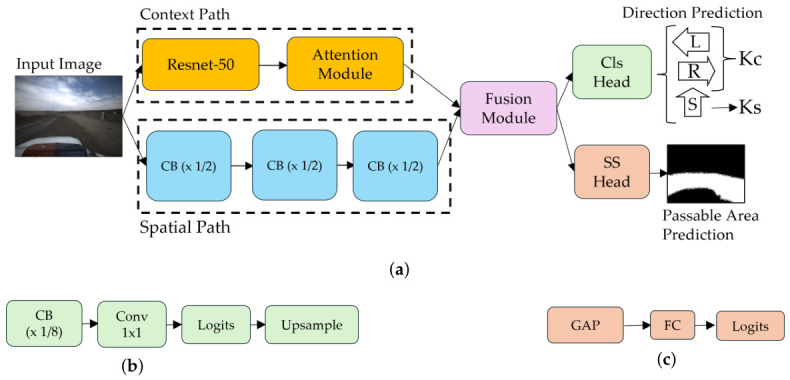
Implemented multitask BiSeNetV1 architecture with a ResNet-50 backbone for drivable-area semantic segmentation and steering-direction classification. The figure represents the complete architecture executed on keyframes: (**a**) Complete multitask architecture, including the Context Path, Spatial Path, Feature Fusion Module, and task-specific output heads. (**b**) Semantic Segmentation head (SS Head), used to predict the drivable-area mask. (**c**) Classification head (Cls Head), used to predict the steering direction. CB denotes convolutional block, GAP denotes Global Average Pooling, and FC denotes Fully Connected layer.

In our implementation, shown in Figure 4, the context path uses ResNet-50 as its backbone, followed by an attention module that refines the extracted semantic features. The spatial path runs in parallel as a stack of convolutional blocks that preserve local geometric information. Both branches converge in the Feature Fusion Module (FFM), which combines the high-level features from the context path with the shallower spatial information. From the fused representation, the network produces two outputs within the same multitask structure: a semantic segmentation head that estimates the drivable area and a classification head that predicts the steering direction. The classification head also yields the control signal that drives the adaptive scheme described in the next subsection.

Let $x_{t}$ be the input image at instant *t*. It is processed in parallel by the spatial and context paths, which yield the representations ${\mathrm{sp}}_{t}\left(\cdot \right)$ and ${\mathrm{ct}}_{t}\left(\cdot \right)$. These two representations are then combined by the fusion module $f_{t}\left(\cdot \right)$, from which the classification output ${\hat{y}}_{t}^{\mathrm{cls}}$ and the segmentation output ${\hat{y}}_{t}^{\mathrm{seg}}$ are produced as:

(5) $${\hat{y}}_{t}^{\mathrm{cls}}=h_{t}^{\mathrm{cls}}\;\left(f_{t}\;\left({\mathrm{sp}}_{t}\left(x_{t}\right),\;{\mathrm{ct}}_{t}\left(x_{t}\right)\right)\right)$$

(6) $${\hat{y}}_{t}^{\mathrm{seg}}=h_{t}^{\mathrm{seg}}\;\left(f_{t}\;\left({\mathrm{sp}}_{t}\left(x_{t}\right),\;{\mathrm{ct}}_{t}\left(x_{t}\right)\right)\right)$$

where $h^{\mathrm{cls}}\left(\cdot \right)$ and $h^{\mathrm{seg}}\left(\cdot \right)$ are the classification and segmentation heads. The same shared representation thus addresses both drivable-area estimation and steering-direction prediction within a single forward pass.

#### 3.2.2. Adaptive Temporal Model

**Figure 5:**
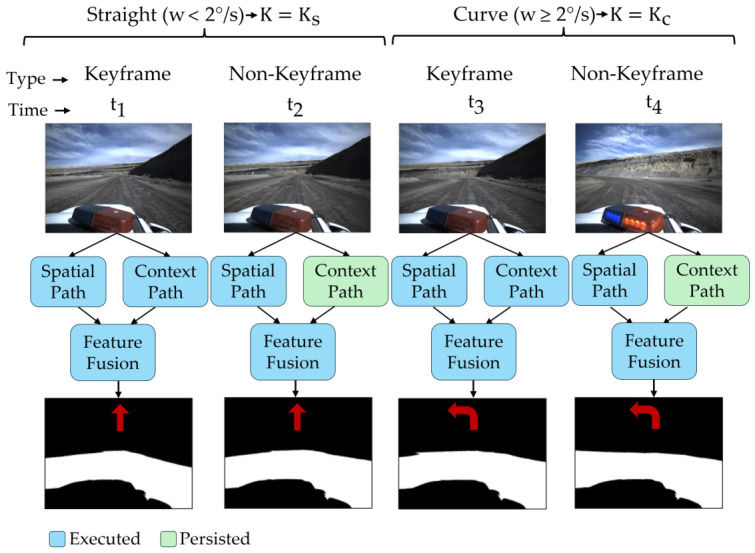
Proposed Adaptive Clockwork (A-CW) inference scheme, in which the classification head controls keyframe selection, the spatial path is executed at every frame, and the context path is updated only on keyframes and reused on non-keyframes.

On top of the multitask architecture, we built an adaptive temporal inference scheme aimed at cutting the cost of frame-by-frame processing. The starting point is a simple observation: consecutive frames of a video sequence share a lot of semantic content, and this is especially true in driving scenes where the trajectory geometry changes only gradually. There is no need, then, to recompute the deep features at every instant: part of the internal representation can be carried across consecutive frames at no perceptual cost. The scheme, sketched in Figure 5, defines two temporal update regimes: a low-frequency one for straight segments, and a higher-frequency one for curves.

Within this scheme, the contextual representation is not refreshed at every frame. Let $t^{*}$ denote the index of the last keyframe available up to instant *t*. The contextual representation used by the model is then

(7) $${\bar{\mathrm{ct}}}_{t}=\left\{\begin{array}{cc} {\mathrm{ct}}_{t}\left(x_{t}\right), & \mathrm{if}\;x_{t}\;\mathrm{is}\;\mathrm{keyframe}\\ {\mathrm{ct}}_{t^{*}}\left(x_{t^{*}}\right), & \mathrm{if}\;x_{t}\;\mathrm{is}\;\mathrm{non}\text{-}\mathrm{keyframe} \end{array}\right.$$

The spatial representation of the current non-keyframe and the contextual representation re-used from the last keyframe are then combined through the feature fusion module:

(8) $${\bar{f}}_{t}=f_{t}\;\left({\mathrm{sp}}_{t}\left(x_{t}\right),\;{\mathrm{ct}}_{t^{*}}\left(x_{t^{*}}\right)\right)$$

The spacing between keyframes is adapted online from the classification output. We define two update periods, $K_{s}$ for straight segments and $K_{c}$ for curved segments, satisfying

(9) $$K_{c}<K_{s}$$

The adaptive policy itself is

(10) $$K=\left\{\begin{array}{cc} K_{c}, & \mathrm{if}\;{\hat{y}}_{t-1}^{\mathrm{cls}}\in \left\{\mathrm{left},\;\mathrm{right}\right\}\\ K_{s}, & \mathrm{if}\;{\hat{y}}_{t-1}^{\mathrm{cls}}=\mathrm{straight} \end{array}\right.$$

When the previous-frame classification flags a turning manoeuvre, the system shortens the keyframe period and refreshes the context branch more often. When it flags a straight segment, the keyframe period is longer and computation is saved.

The specific values of $K_{c}$ and $K_{s}$ were not fixed a priori but determined experimentally from a grid search over the range [10, 100] frames, which corresponds to context-path refresh intervals of approximately 1–10 s at the 10 FPS recording rate used by the dataset. This range was chosen on practical grounds: updating the context path more often than every 1 s provides limited benefit given the slow visual change rate of mining haul roads, while leaving the context path frozen for longer than 10 s risks accumulating stale deep features through multiple geometric transitions. The selected configuration ($K_{c}$ = 10, $K_{s}\;=\;100$) emerged as the best trade-off between classification accuracy, segmentation performance, and GPU-only inference speed across both evaluation routes; the full grid-search results are reported in Appendix B.

### 3.3. Model Training Process

Datasets for semantic segmentation and visual perception typically contain thousands of annotated samples, a clear indication of the cost of manual acquisition and labelling in this domain [29,67]. We had only 100 labelled images from the source domain, so we resorted to dynamic data augmentation to compensate for the limited supervision and improve model robustness. The original images had a resolution of 2048×1536 pixels and were resized to 512×512 pixels for training. The multitask model was trained on isolated images, without explicitly exploiting any temporal relationship between consecutive frames. Training ran for 40,000 iterations with a batch size of 4, which amounts to 160,000 training exposures. Because the training set contained only 100 manually annotated frames, overfitting was considered a relevant risk. To mitigate this, the augmentation pipeline was applied on the fly at every iteration: augmented samples were not stored as a pre-computed expanded dataset but generated dynamically during training. This exposed the model to geometric and photometric variations at each iteration, increasing the effective variability of the training process and limiting direct memorisation of a fixed set of augmented images. This strategy does not, however, remove the limitation imposed by the small annotated dataset; accordingly, the reported robustness should be interpreted within the scope of the available mining sequences and the evaluated protocol.

For validation during training, we built a static set of 20 reserved original images and generated 60 additional augmented samples from them, giving a validation subset of 80 images.

When a horizontal-flip transformation was applied to an image taken on a curve, the classification label was adjusted to remain consistent with the symmetry of the scene: samples labelled as right turn were re-labelled as left turn, and vice versa.

The augmentation parameters used during training and validation are listed in Table 2.

#### 3.3.1. Multitask Loss Function

Both the binary semantic segmentation task and the steering-direction classification task were trained with a Cross-Entropy (CE) loss. The total loss is the weighted linear combination of the two task losses, Equation (11):

(11) $$L=\Delta_{\mathrm{seg}}\;L_{\mathrm{seg}}+\Delta_{\mathrm{cls}}\;L_{\mathrm{cls}}$$

where $L_{\mathrm{seg}}$ and $L_{\mathrm{cls}}$ are the segmentation and classification losses, and $\Delta_{\mathrm{seg}}$ and $\Delta_{\mathrm{cls}}$ are weighting constants fixed at 0.5 and 1, respectively. The individual losses read:

(12) $$L_{\mathrm{seg}}=\frac{1}{N}\sum_{i=1}^{N}\mathrm{CE}\;\left({\hat{y}}_{i}^{\mathrm{seg}},\;y_{i}^{\mathrm{seg}}\right)$$

(13) $$L_{\mathrm{cls}}=\frac{1}{N}\sum_{i=1}^{N}\mathrm{CE}\;\left({\hat{y}}_{i}^{\mathrm{cls}},\;y_{i}^{\mathrm{cls}}\right)$$

where *N* is the number of samples in a single iteration, ${\hat{y}}_{i}^{\mathrm{seg}}$ and ${\hat{y}}_{i}^{\mathrm{cls}}$ are the model predictions for sample *i*, and $y_{i}^{\mathrm{seg}}$ and $y_{i}^{\mathrm{cls}}$ are the corresponding reference annotations.

Semantic segmentation operates on two classes, drivable area and environment. Classification operates on three classes describing the direction of travel: left, straight, and right.

#### 3.3.2. Training Implementation

The multitask model was trained within MMSegmentation on images resized to 512×512 pixels. The context branch of BiSeNetV1 was initialised with a ResNet-50 pretrained on ImageNet.

Training ran on a single NVIDIA A100 80 GB GPU. Validation was performed every 200 iterations, computing mIoU for semantic segmentation and Top-1 Accuracy for classification. Checkpoints were stored according to the best performance observed on each task. The main hyperparameters are summarised in Table 3.

### 3.4. Implementation of Temporal Models Based on Multitask BiSeNet

To compare different temporal optimisation strategies for video inference, we implemented two temporal variants of the multitask BiSeNetV1 model: one based on Deep Feature Flow (DFF) [68], with a fixed update schedule, and one based on the Low-Latency Method (LLM) [60], with an adaptive keyframe-selection policy.

All temporal variants preserved the same multitask output structure of the proposed model. Each method therefore produced both drivable-area segmentation logits and steering-direction classification logits. The segmentation and classification heads were kept consistent with the base multitask BiSeNet model, while the temporal modules modified how intermediate features were updated, propagated, or reused across consecutive frames. This allowed the comparison to focus on the temporal inference strategy rather than on changes in the task heads.

Both temporal models were trained on video sequences from Route A. Unlike the proposed Adaptive Clockwork model—trained on isolated images—the DFF and LLM variants use temporal information during training. For this reason, the training samples were organised as temporal pairs or short sequences, depending on the requirements of each method. We kept a comparable family of augmentations, adapted to the sequential or paired input format each method requires. Appendix C summarises the main implementation differences between Adaptive Clockwork and the DFF and LLM temporal variants.

The DFF variant is trained on sequences made of keyframes and non-keyframes. Keyframes were processed by the full multitask BiSeNet model to obtain updated features and task predictions. For non-keyframes, an optical-flow network based on FlowNetC was added to estimate the temporal correspondence between the keyframe and the current frame. The estimated flow was then used to propagate the feature representation from the keyframe to the current frame, reducing the need to recompute the complete network at every instant. The mapping between the original DFF components and our BiSeNet-based implementation is given in Appendix D.

The LLM variant is trained in two stages: the adaptive propagation module is first optimised starting from the multitask BiSeNet checkpoint with the best classification performance; then, the scheduler is trained to estimate the temporal deviation between the keyframe and the current frame. This first stage optimises the temporal feature propagation mechanism used to process non-keyframes, while the second stage trains the scheduler to determine whether the current frame should be processed as a new keyframe or as a non-keyframe. At inference time, the scheduler decides whether a frame is a keyframe or a non-keyframe according to an external threshold τ fixed at evaluation. Appendix E maps LLM onto its BiSeNet-based counterpart.

These implementations provide two reference temporal strategies with different update mechanisms. DFF uses a fixed keyframe schedule with optical-flow-based feature propagation, whereas LLM uses a learned scheduler to determine the keyframe update online. They were evaluated together with Fixed Clockwork, Adaptive Clockwork, and the frame-by-frame multitask baseline under the protocol described in the following section.

### 3.5. Model Evaluation Protocol

Model evaluation was structured in three stages. First, during training and model selection, the final checkpoint was selected on the validation set according to classification performance, since the classification output is both a driver-assistance cue and the control signal for the adaptive temporal policy. Second, the selected BC checkpoint was evaluated on the temporal test routes A and B to measure drivable-area segmentation and steering-direction classification performance along complete onboard sequences. Third, the temporal inference strategies were evaluated inside the simulated ADAS pipeline, where processing includes preprocessing, data transfer, inference, prediction transfer, and CPU-side visual postprocessing for the HMI output. Every inference benchmark was run on the same NVIDIA A100 80 GB GPU; the computational environment was therefore kept consistent across all compared methods.

The proposed model was compared against a BiSeNet frame-by-frame baseline and against temporal strategies obtained by adapting the multitask BiSeNet architecture. We evaluated Fixed Clockwork, Deep Feature Flow (DFF), and Low-Latency Method (LLM) configurations. The evaluated keyframe periods were defined between 10 and 100 frames. Since the source sequences were recorded at 10 FPS, this range corresponds approximately to context-path refresh intervals between 1 s and 10 s. These values were chosen to cover configurations with relatively frequent context updates as well as those with stronger temporal reuse.

From these configurations, the Adaptive Clockwork strategy used in the focused evaluations was selected according to the best trade-off between steering-direction classification accuracy, drivable-area segmentation performance, and GPU-only inference speed. Since the classification output also controls the adaptive keyframe policy, classification performance was considered the primary selection criterion.

#### 3.5.1. Model Inference Performance

Because the classification output plays a double role in our proposal—as a perceptual driver-assistance output and as the control signal for the adaptive temporal scheme—we selected the final checkpoint based on classification performance on the validation set. Concretely, we picked the checkpoint with the highest Top-1 Accuracy on the validation set, given that this task directly conditions the temporal update policy. The validation results were used solely for checkpoint selection; the subsequent temporal evaluation on Routes A and B was performed independently using the selected BC checkpoint.

Performance was evaluated on the two tasks solved by the multitask architecture. We used mean Intersection over Union (mIoU) for drivable-area semantic segmentation and Top-1 Accuracy for steering-direction classification. Both metrics were computed over the two test sequences described earlier, which lets us inspect the behaviour of the model under different driving scenarios within the mining environment.

For the segmentation task, mIoU is defined as the average overlap between the model prediction and the reference annotation across classes. With $C_{\mathrm{seg}}$ segmentation classes:

(14) $$\mathrm{mIoU}=\frac{1}{C_{\mathrm{seg}}}\sum_{c=1}^{C_{\mathrm{seg}}}\frac{\left|{\hat{y}}_{c}^{\mathrm{seg}}\cap y_{c}^{\mathrm{seg}}\right|}{\left|{\hat{y}}_{c}^{\mathrm{seg}}\cup y_{c}^{\mathrm{seg}}\right|}$$

where ${\hat{y}}_{c}^{\mathrm{seg}}$ is the set of pixels predicted by the model as belonging to class *c*, and $y_{c}^{\mathrm{seg}}$ is the set of pixels labelled with that class in the reference. We use $C_{\mathrm{seg}}=2$, with classes drivable area and environment.

For the classification task, we use Top-1 Accuracy, defined as:

(15) $$\mathrm{Top}\text{-}1\;\mathrm{Acc}=\frac{1}{N_{\mathrm{eval}}}\sum_{i=1}^{N_{\mathrm{eval}}}I\;\left\{{\hat{y}}_{i}^{\mathrm{cls}}=y_{i}^{\mathrm{cls}}\right\}$$

where $N_{\mathrm{eval}}$ is the total number of evaluated samples, ${\hat{y}}_{i}^{\mathrm{cls}}$ is the class predicted for sample *i*, $y_{i}^{\mathrm{cls}}$ is the corresponding ground-truth label, and I is the indicator function, returning 1 when the condition is met and 0 otherwise.

#### 3.5.2. Model Inference Speed Measurement

This experiment measures model inference speed in isolation, with the sole purpose of quantifying how long the network takes to produce its predictions. In this work, this isolated speed measurement was used during the validation and model-selection stage, where the evaluated models were processed in frame-by-frame mode on the validation set. From the average per-frame time, the inference speed in FPS follows as:

(16) $${\mathrm{FPS}}_{\mathrm{GPU}}=\frac{1000}{{\bar{t}}_{\mathrm{GPU}}}$$

where ${\bar{t}}_{\mathrm{GPU}}$ is the average inference time per frame in milliseconds.

#### 3.5.3. ADAS Simulation

**Figure 6:**
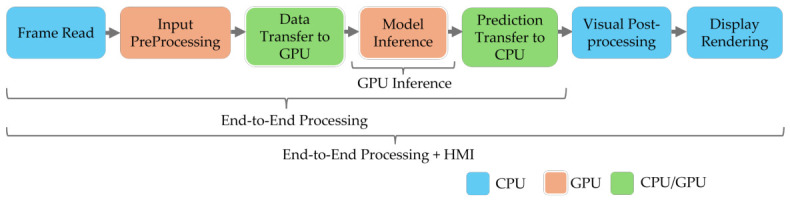
Processing pipeline of the simulated ADAS system used for inference-speed evaluation. The brackets indicate the stages included in each FPS measurement mode. Each frame is read from the input sequence, preprocessed, transferred to the GPU for model inference, and then returned to the CPU for visual post-processing and HMI overlay generation. The final display-rendering stage simulates a 1080×720 RGB HMI output fixed at 10 FPS and is not included in the FPS measurements.

For the ADAS simulation, we followed the processing pipeline shown in Figure 6. Each frame is read from the input sequence and then preprocessed into the network input. The preprocessed frame is transferred to the GPU, where inference takes place. The outputs are then sent back to the CPU for visual postprocessing: the drivable-area mask is overlaid on the original frame and the predicted steering-direction label is drawn at the top centre of the image. The final HMI output is rendered on a simulated RGB display of 1080×720 pixels.

Processing speed is reported under two measurement modes: (i) GPU Inference, i.e., the time taken by the model on the GPU, and (ii) End-to-End Processing, which covers input preprocessing, data transfer, inference, prediction transfer, and CPU-side visual postprocessing. The final screen-rendering stage is not part of the FPS measurement, as the simulated HMI is fixed at 10 FPS.

### 3.6. Selected Adaptive Strategy and Focused Evaluations

Following the general evaluation protocol described in Section 3.5, the Adaptive Clockwork configuration that offered the best trade-off between steering-direction classification accuracy, drivable-area segmentation performance, and GPU-only inference speed was selected for a set of focused evaluations. This section describes those evaluations, whose shared purpose is to characterise the contribution, the computational behaviour, and the robustness of the selected strategy under varied conditions.

The focused evaluation begins with a progressive ablation of the adaptive strategy, comparing the multitask frame-by-frame baseline, Fixed Clockwork, and the selected Adaptive Clockwork configuration. A comparison with dedicated video segmentation models is then included, since the proposed architecture builds on an efficient semantic segmentation backbone and its primary perceptual output is the traversable-area mask; this comparison provides an external reference against segmentation-oriented alternatives while keeping the multitask nature of the method explicit.

Finally, the selected strategy is assessed in three additional scenarios: execution on different GPU platforms using the simulated ADAS pipeline, adaptive inference under high semantic redundancy, and robustness against synthetic visual degradations.

#### 3.6.1. Progressive Ablation of the Adaptive Strategy

A progressive ablation study was conducted to quantify the contribution of each step leading to the selected Adaptive Clockwork configuration. The goal was to observe how perceptual performance and inference speed change as the system moves from multitask frame-by-frame inference, through a fixed temporal re-use strategy, and finally to an adaptive policy guided by the classification output.

The comparison was carried out on Routes A and B, reporting Top-1 Accuracy, road-class IoU, and GPU-only FPS.

#### 3.6.2. Comparison with Video Segmentation Models

The selected strategy was compared with representative semantic segmentation baselines for images and video. The purpose was to position the perceptual performance and computational efficiency of the proposed method relative to alternatives in this task.

Mask2Former [69] was chosen as the frame-by-frame semantic segmentation baseline, representing a modern mask-prediction architecture widely adopted as a reference in semantic segmentation benchmarks. Additionally, MPVSS [70] was evaluated; it adapts Mask2Former for efficient video semantic segmentation by applying a fixed-interval keyframe update scheme. Including MPVSS enables examination of the effect of introducing temporal processing on a more complex segmentation architecture than BiSeNet while excluding the classification-guided adaptive policy.

To maintain comparable conditions, all models were evaluated on Routes A and B using the same input resolution and a ResNet-50 backbone. Temporal training of MPVSS followed a procedure analogous to that used for the other sequence-based methods. The comparison covered both segmentation performance and computational complexity.

#### 3.6.3. Hardware Benchmark for the Simulated ADAS Pipeline

The selected Adaptive Clockwork strategy was evaluated on multiple GPU platforms using the same simulated ADAS pipeline protocol described in Section 3.5.3. The purpose of this benchmark was to characterise the system’s sensitivity to available hardware and to provide a reference for deployments on computationally constrained platforms.

To enable execution across different hardware, the selected model was converted to ONNX format. This conversion produced a portable model representation and preserved an equivalent inference flow during the hardware tests. All benchmarks were run with a batch size of 1, consistent with online single-frame processing. A warm-up phase of 100 inference passes was applied on each platform before recording measurements, so that GPU clocks and memory allocation reached steady state. Inference was performed in FP32 precision throughout, and no quantisation was applied.

The evaluated platforms were an NVIDIA RTX 2000 Ada, an NVIDIA L4, and an NVIDIA A100 PCIe. The RTX 2000 Ada and the L4 served as lower-power hardware references, while the A100 PCIe represented a data-centre GPU baseline. The main specifications of these platforms are summarised in Appendix F.

For each platform, average end-to-end latency, end-to-end FPS, GPU-only FPS, and GPU memory used during the simulated pipeline were reported. These results characterise the perception-pipeline throughput on the evaluated GPU platforms and should not be interpreted as end-to-end performance on an embedded onboard system, since no embedded hardware deployment was conducted.

#### 3.6.4. Adaptive Inference Under High Semantic Redundancy

An operational experiment was designed to analyse the benefit of the selected strategy under conditions of high semantic redundancy. Such a situation arises, for instance, when the vehicle remains stationary and the scene observed by the camera changes very little between consecutive frames. In this context, frequent updates of deep contextual features are not strictly necessary, so an adaptive policy can reduce accumulated GPU computation time compared with frame-by-frame inference without compromising perceptual output.

The simulation placed the vehicle stopped on a straight section at the beginning of Route B and kept the ADAS perception pipeline active. The experiment was run on an NVIDIA A100 PCIe GPU and compared the selected Adaptive Clockwork configuration against two references: multitask frame-by-frame inference and Fixed Clockwork. For each method, cumulative GPU-only time was estimated over different stop durations, using the per-route average inference time per frame obtained during the main evaluation. From this measurement, the cumulative difference between Adaptive Clockwork and each baseline was computed, quantifying how GPU-only time savings grow as scene temporal redundancy increases.

#### 3.6.5. Robustness Under Visual Degradations

The robustness of the selected strategy was evaluated by applying synthetic visual degradations to the test sequences of Routes A and B, without retraining the model. The objective was to assess whether the system maintains adequate inference performance under adverse visual conditions plausible in open-pit mining operations.

Degraded sequences were generated by applying image-processing filters and transformations to the original evaluation frames. These transformations alter the visual appearance of the scene while preserving its geometric structure and the original annotations, allowing the same segmentation and classification references to be used throughout the evaluation.

**Figure 7:**
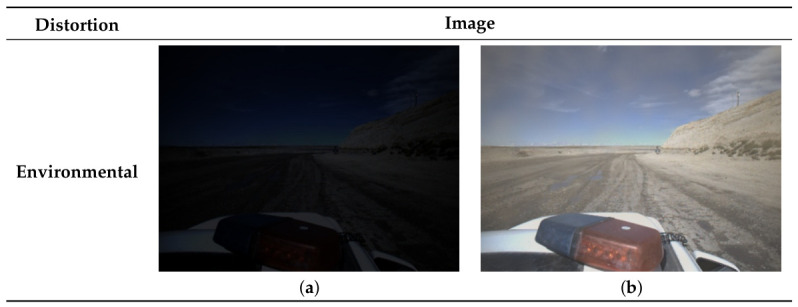

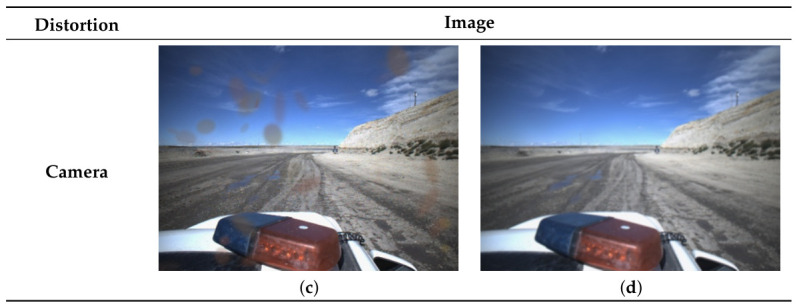
Representative examples of the visual degradations used in the robustness evaluation. The evaluated degradations include environmental conditions, such as (**a**) luminosity reduction and (**b**) suspended dust, and camera-related conditions, such as (**c**) mud occlusion and (**d**) defocus blur.

The degradations were organised into two categories. The first covers environmental conditions, namely luminosity changes and suspended dust. The second covers camera-related conditions, including mud or dirt occlusion on the windshield and defocus blur. Each degradation was applied at two severity levels, defined by a scalar parameter specific to each transformation. Figure 7 shows representative examples of the evaluated degradations.

Degraded sequences were processed using the same inference protocol applied to the original sequences. Performance was reported for Routes A and B using road-class IoU and Top-1 classification Accuracy.

## 4. Results

We report the experimental results for the multitask model and for the temporal inference strategies evaluated against it. The section is structured in four main parts: training and model selection; temporal feature variation; ADAS video sequence evaluation of temporal inference strategies; and focused evaluations of the selected Adaptive Clockwork configuration.

### 4.1. Training and Model Selection

**Figure 8:**
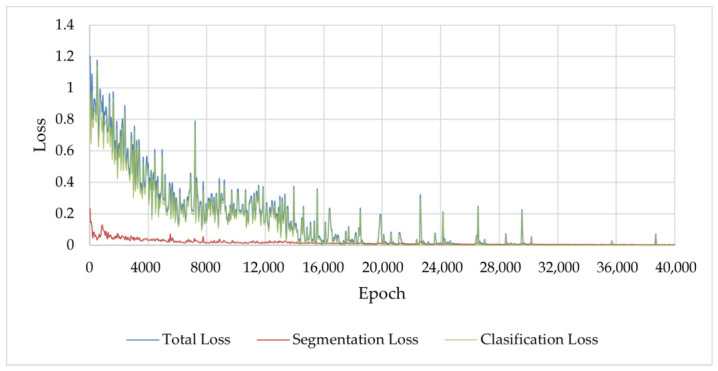
Training curves of the proposed multitask model. The dashed vertical lines indicate the selected checkpoints: Best Classification (BC) at iteration 25,600 and Best Segmentation (BS) at iteration 33,400.

Figure 8 traces the evolution of the total loss and of the segmentation and classification losses during training of the multitask model. Dashed vertical lines mark the two checkpoints retained for downstream use: Best Classification (BC) and Best Segmentation (BS).

Table 4 reports the validation performance of the multitask BiSeNet model alongside the single-task classification and segmentation variants. The evaluation was performed frame-by-frame on the validation set used during training. For the multitask model, we report both BC and BS, while for the single-task variants, we report the checkpoint chosen for each task. The table includes Top-1 Accuracy, mIoU, FPS, and parameter count.

Since the classification head plays a double role in our proposal—ADAS perceptual output and control signal for the adaptive keyframe policy—all temporal experiments were run with the BC checkpoint, which was selected as the checkpoint with the highest classification performance on the validation set.

### 4.2. Temporal Feature Variation

**Figure 9:**
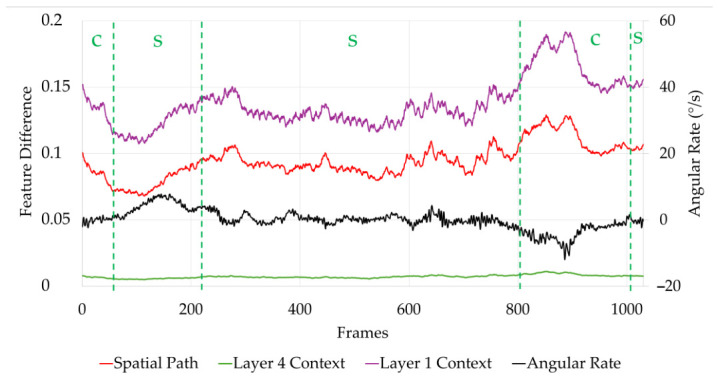
L1-norm-based feature differences between consecutive frames for different layers of the BiSeNet model on Route A. The curves compare the temporal variation of Spatial Path and Context Path features with the vehicle angular rate. The letters S and C indicate straight and curved trajectory sections, respectively.

Figure 9 reports the difference between features extracted from consecutive frames on Route A, measured with the L1 norm. The curves correspond to different levels of the BiSeNet architecture: the Spatial Path, a shallow layer (Layer 1) and a deep contextual layer (Layer 4) of the Context Path. The vehicle angular rate is also plotted, and the straight and curved sections of the trajectory are marked with the letters S and C.

Figure 9 shows the temporal evolution of the model’s internal representations along the evaluated sequence side by side with the angular-rate variation that accompanies trajectory changes.

### 4.3. ADAS Video Sequence Evaluation of Temporal Inference Strategies

This section presents the evaluation of the temporal inference strategies on the complete video sequences used in the ADAS simulation. The analysis covers Routes A and B and reports classification accuracy, frame-level segmentation behaviour, simulated pipeline processing speed, and qualitative examples of the system’s visual outputs. Taken together, these results allow the strategies to be compared not only on per-frame performance metrics but also on their behaviour during continuous video processing.

#### 4.3.1. Classification Performance and GPU-Only Speed

**Figure 10:**
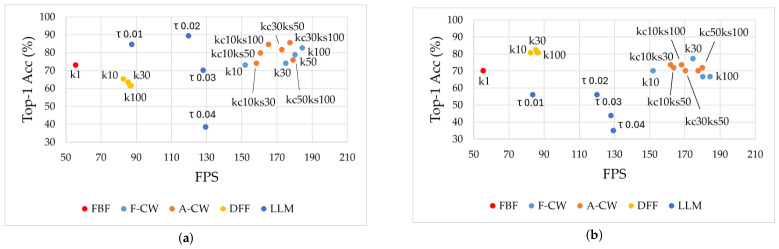
Top-1 Accuracy versus FPS for the evaluated inference methods using the BC checkpoint. The temporal strategies were evaluated by adapting the proposed multitask BiSeNet architecture: (**a**) Route A. (**b**) Route B. FBF, F-CW, A-CW, DFF, and LLM denote Frame-by-Frame Evaluation, Fixed Clockwork, Adaptive Clockwork, Deep Feature Flow, and Low Latency Method, respectively.

Figure 10 plots Top-1 Accuracy against GPU-only FPS for the inference strategies evaluated under checkpoint BC. Results are reported separately for Route A and Route B. The figure includes the BiSeNet frame-by-frame model, Fixed Clockwork, Adaptive Clockwork, DFF, and Low-Latency Method (LLM), all of them implemented as adaptations of our multitask BiSeNet architecture.

Each point in Figure 10 corresponds to a specific configuration of the method it belongs to. The horizontal axis is GPU inference speed and the vertical axis is classification performance measured with Top-1 Accuracy; for each configuration, accuracy and speed can therefore be read off jointly. Appendix G contains the confusion matrices of the best-performing configurations of each method.

#### 4.3.2. Frame-Level mIoU Distribution

**Figure 11:**
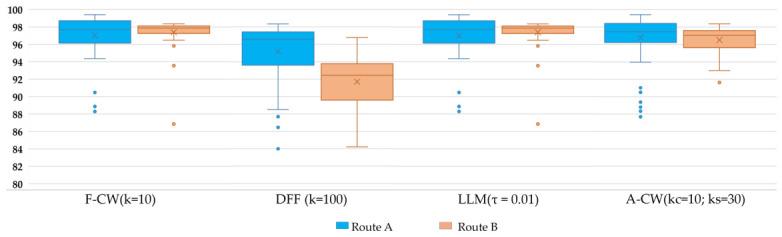
Boxplot comparison of frame-level mIoU for the selected temporal inference strategies on Routes A and B. The plotted methods correspond to the best segmentation configuration selected from each evaluated method family. F-CW, DFF, LLM, and A-CW denote Fixed Clockwork, Deep Feature Flow, Low-Latency Method, and Adaptive Clockwork, respectively. In each boxplot, the central line indicates the median, the cross indicates the mean, the box represents the interquartile range, the whiskers show the data range excluding outliers, and the points indicate outlier frames.

Figure 11 shows boxplots of the frame-level mIoU distribution for a subset of temporal inference strategies on Routes A and B. Within each method family, we selected the configuration with the best segmentation inference performance. Blue boxes correspond to Route A and orange boxes to Route B. In every case, the boxplot summarises the distribution of frame-level mIoU values, including median, mean, interquartile range, and outliers.

#### 4.3.3. Processing Throughput in the Simulated ADAS Pipeline

Table 5 and Table 6 summarise processing speed and keyframe usage for the methods evaluated on Routes A and B. Both tables report two measurement modes: GPU-only FPS, i.e., the GPU inference time of the model, and display-excluded end-to-end (E2E) processing, which covers the preprocessing, data transfer, inference, prediction transfer, and visual postprocessing pipeline. The number of keyframes and non-keyframes processed by each strategy is also provided.

Table 5 reports the results of the BiSeNet frame-by-frame model and of the configurations with a fixed schedule. Table 6 reports the adaptive-policy methods, namely Adaptive Clockwork (A-CW) and the Low Latency Method (LLM).

#### 4.3.4. Visual Outputs from the Simulated ADAS Pipeline

Table 7 and Table 8 show qualitative examples of the outputs produced by the evaluated temporal inference methods. Table 7 corresponds to Route A and Table 8 to Route B. For each method family, we display the configuration selected according to the inference performance criterion used in the evaluation.

In both tables, each column corresponds to a temporal inference method and each row to a time instant of the sequence. The red overlay represents the predicted drivable area, and the cyan arrow indicates the steering direction estimated by the classification head.

### 4.4. Selected Adaptive Strategy and Focused Evaluations

Following the general evaluation of the temporal inference strategies, the Adaptive Clockwork configuration with $k_{c}=10$ and $k_{s}\;=\;100$ was selected as the best trade-off across classification accuracy, segmentation performance, and GPU-only speed. Since the classification output also controls the adaptive keyframe-selection policy, particular emphasis was placed on classification performance when selecting the final configuration. This section presents the results obtained for that configuration across five studies: progressive ablation, comparison with video segmentation models, hardware benchmark, inference under high semantic redundancy, and robustness under visual degradations.

#### 4.4.1. Progressive Ablation Study of the Adaptive Strategy

Table 9 presents the results of the progressive ablation study of the selected adaptive strategy.

#### 4.4.2. Comparison with Video Segmentation Models

**Figure 12:**
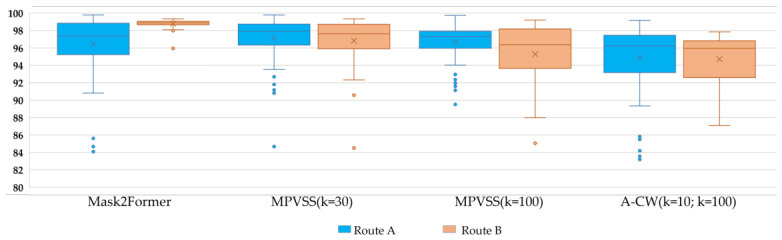
Frame-level road-class IoU distribution for video segmentation models and the selected A-CW strategy.

Figure 12 and Table 10 present the comparison between the selected A-CW strategy and the evaluated segmentation models. The comparison includes Mask2Former, MPVSS (*k* = 30), MPVSS (*k* = 100), and A-CW ($k_{c}=10$, $k_{s}$ = 100), covering the mIoU distribution, parameter count, and GPU-only speed on Routes A and B.

#### 4.4.3. Hardware Benchmark of the Simulated ADAS Pipeline

Table 11 presents the hardware benchmark results of the simulated ADAS pipeline for the selected A-CW strategy. The evaluated platforms were an NVIDIA RTX 2000 Ada, an NVIDIA L4, and an NVIDIA A100 PCIe. For each platform, average end-to-end latency, end-to-end FPS, GPU-only FPS, and GPU memory usage are reported.

#### 4.4.4. Adaptive Inference Under High Semantic Redundancy

**Figure 13:**
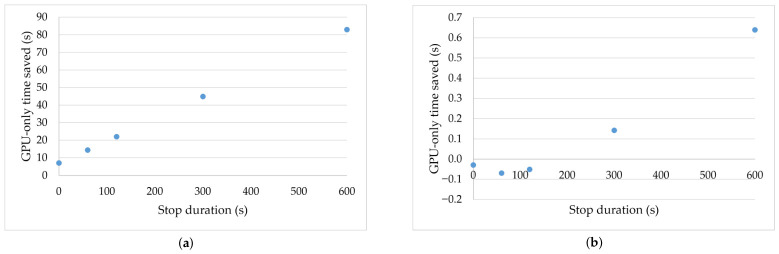
Cumulative GPU-only time saving under high semantic redundancy. (**a**) Difference between A-CW and multitask frame-by-frame inference (*k* = 1). (**b**) Difference between A-CW and Fixed Clockwork (*k* = 50).

Figure 13 shows the cumulative GPU-only time savings obtained in the high semantic redundancy scenario. The experiment compares the selected A-CW strategy against two references: multitask frame-by-frame inference and Fixed Clockwork. Figure 13a shows the cumulative difference between A-CW ($k_{c}=10$, $k_{s}$ = 100) and frame-by-frame inference across the evaluated stop durations. Figure 13b shows the cumulative difference between A-CW ($k_{c}=10$, $k_{s}$ = 100) and Fixed Clockwork under the same stop intervals.

In both plots, the horizontal axis represents the simulated stop duration and the vertical axis represents the accumulated GPU-only time saving. Results are reported for the simulated scenario on Route B.

#### 4.4.5. Robustness Under Visual Degradations

Table 12 and Table 13 present the robustness results of the selected A-CW strategy under synthetic visual degradations. The degradations are grouped into environmental conditions and camera-related conditions. For each condition, road-class IoU and Top-1 Accuracy are reported for Routes A and B.

## 5. Discussion

The experiments support the central design choice of this paper: that drivable-area segmentation and steering-direction classification can be accommodated within a single multitask network without degrading the performance of either task. This matters most acutely in mining, where labelled data is scarce and producing semantic masks requires hours of manual annotation per scene. Rather than maintaining two independent models, the approach re-uses one shared backbone to serve both outputs simultaneously.

A closer inspection of the validation results confirms that the classification head does not meaningfully hurt segmentation. In Table 4, the multitask network reaches 97.33% mIoU at checkpoint BC, against 98.72% for the segmentation-only baseline—a difference of well under two percentage points. Top-1 Accuracy, by contrast, climbs from 83.75% in the classification-only model to 96.25% in the multitask variant. The shared representation evidently balances fine spatial detail with the global information needed to identify the steering direction, and it achieves this without compromising segmentation performance.

The temporal feature-variation analysis in Figure 9 adds a piece of evidence that the model’s internal representations do not vary uniformly along the sequence. Variability spikes when something perceptually significant happens—the start of a curve, typically. This is also the hypothesis the Adaptive Clockwork scheme relies on: not every frame deserves the same contextual update frequency. On straight sections, the scene evolves slowly and feature re-use saves work; on curves, where trajectory geometry and perspective change quickly, refreshing the deep features more often is worth it.

At inference time, segmentation mIoU stays within a high range across the evaluated strategies on both Route A and Route B. The frame-level mIoU distribution confirms that the selected configurations preserve stable segmentation even when temporal re-use or propagation is introduced. Top-1 Accuracy is more sensitive: it varies more across strategies. That sensitivity is relevant in our system because the classification task is, simultaneously, the driver-assistance output and the controller of the adaptive update policy.

The two evaluation routes provide complementary conditions for interpreting the temporal results. Route A presents higher trajectory variability, with both right- and left-hand curves, and partially shares its source scenario with the training set. Route B is shorter and geometrically simpler but was kept outside the training source entirely, making it a more demanding context in which to assess generalisation. Its slightly lower mIoU and Top-1 Accuracy values suggest that the absence of source overlap introduces a more challenging evaluation condition despite the simpler trajectory geometry.

The progressive ablation of the selected strategy further separates the effect of the main design steps. The frame-by-frame multitask configuration provides the reference case without any temporal re-use. Fixed Clockwork introduces context-path re-use with a constant update period, while Adaptive Clockwork adds classification-guided keyframe selection on top of that re-use mechanism. This progression increases inference efficiency while maintaining segmentation performance and even improving classification accuracy, demonstrating that the two components are complementary rather than competing.

In terms of efficiency, the temporal strategies push GPU-only throughput well above the frame-by-frame baseline. In the simulated ADAS evaluation, Adaptive Clockwork with $k_{c}$ = 10 and $k_{s}$ = 100 reaches 165.19 FPS on Route A and 168.01 FPS on Route B, against 55.54 and 55.33 FPS for the frame-by-frame baseline. Selective feature re-use cuts the inference cost of the model while keeping the multitask architecture as a shared perceptual core, with no changes to the network itself.

The comparison with Fixed Clockwork brings out the value of a scene-dependent update policy. Under a fixed interval, the spacing between keyframes is constant along the sequence regardless of whether the vehicle is on a straight or in the middle of a curve. Adaptive Clockwork modulates that update frequency from the steering-direction classification, and this matters because the classification task is doing more than producing a driver-facing cue: it is also flagging scene conditions of higher variability. The improvement in classification behaviour observed under A-CW can be read as the consequence of a contextual update that tracks trajectory dynamics more closely, especially in curves.

DFF and LLM differ from our scheme along more than just accuracy and speed. Methodologically, DFF propagates features through optical flow and therefore adds a dedicated module to estimate the temporal correspondence between keyframe and current frame. In our experiments, DFF reached lower GPU-only speeds than Clockwork, which suggests that, for this architecture and scenario, explicit optical-flow propagation is more costly than direct contextual feature re-use. LLM adds adaptive propagation plus a trained scheduler, and its behaviour depends on a threshold τ fixed at evaluation time. Adaptive Clockwork sidesteps both: no extra temporal training and no external scheduler module—the control signal comes straight from the classification output, which had to be computed anyway as part of the ADAS interface.

The comparison with Mask2Former and MPVSS provides an additional reference against segmentation-oriented models. Mask2Former represents a strong frame-by-frame semantic segmentation baseline, while MPVSS introduces fixed temporal updates for video semantic segmentation. Against both of these, the selected A-CW configuration achieves higher GPU-only throughput while preserving the full multitask ADAS output.

The ADAS simulation makes one practical point clear. The gap between GPU-only FPS and display-excluded E2E FPS shows that the speed gain at the inference stage does not translate linearly into operational throughput. Comparing Adaptive Clockwork with the BiSeNet frame-by-frame baseline on Route A, GPU-only speed climbs from 55.54 to 165.19 FPS (about 3.00×), while E2E speed climbs from 26.18 to 38.12 FPS (about 1.47×). Route B behaves similarly: GPU-only goes from 55.33 to 168.01 FPS (∼3.03×), E2E from 25.73 to 37.51 FPS (∼1.45×). The explanation is mundane but important. Adaptive Clockwork reduces inference cost by re-using features on non-keyframes, but preprocessing, data transfer and visual postprocessing keep running on every frame. End-to-end throughput therefore depends on both the temporal inference strategy and the common stages of the perception–HMI pipeline.

The hardware benchmark extends this point across different GPU platforms. The RTX 2000 Ada and NVIDIA L4 experiments provide lower-power references closer to edge-oriented execution. The selected A-CW strategy achieves lower throughput on these lower-power platforms but still satisfies the 10 FPS requirement of the simulated HMI in both GPU-only and E2E measurements across all evaluated platforms. These results should nonetheless be understood as perception-pipeline benchmarks rather than a complete onboard deployment inside a mining truck.

The high-semantic-redundancy experiment adds an operational view of the adaptive policy. When the vehicle remains stopped on a straight segment, consecutive frames provide limited new contextual information. Under this condition, Adaptive Clockwork accumulates GPU-only time savings relative to frame-by-frame inference while retaining the ability to shorten the update period whenever the predicted driving condition changes.

The robustness evaluation shows that the selected strategy does not respond equally to all visual degradations. Dust and mud-like occlusions produced limited changes in road-class IoU, whereas severe luminosity reduction and strong defocus caused larger performance drops. Dust and mud occlusions typically affect only localised image regions, allowing the network to preserve sufficient contextual and structural cues for road segmentation. Severe luminosity reduction and strong defocus, by contrast, alter the visual information across most or all of the image, degrading the edge definition, texture patterns, and contrast that are important for both segmentation and classification. Since the degradations were synthetically generated, these results should be regarded as a controlled sensitivity analysis rather than a complete validation under real adverse environmental conditions.

Read from the angle of vehicular onboard sensing, the results indicate that lightweight multitask architectures can reliably interpret in-cab imagery from a moving truck in unstructured industrial sites with limited labelled data, reaching competitive segmentation accuracy (mIoU >95.9%) and real-time speeds (>165.19 FPS GPU-only) under the Adaptive Clockwork scheme. The point is not a minor one: most state-of-the-art models for outdoor scene perception have been benchmarked on urban driving datasets, whose sensing geometry, scene statistics and operational constraints differ noticeably from those of open-pit mining. The classification-guided temporal update policy proposed here is a practical route towards efficient onboard perception in mining without densely annotated video—one of the main obstacles to building datasets in this kind of industrial setting. More broadly, the idea of coupling the temporal update of a perception backbone to a lightweight, semantically meaningful classification signal should carry over to other onboard sensing platforms—ground or aerial—where dense per-frame annotation is not available but a coarse contextual cue (trajectory regime, illumination state, weather class) can be obtained online.

Taken together, these observations suggest the approach is an efficient and interpretable option for multitask video perception in mining. Its main contribution goes beyond the raw inference speedup: the temporal update policy is driven by an output that is itself useful to the ADAS. Steering-direction classification helps the driver and, at the same time, governs the computational cost of the perception backbone during sequential inference.

A final limitation concerns the size of the annotated training set. Although online data augmentation increased the effective variability of the training process, the model was still trained from only 100 manually annotated frames. Overfitting to the limited source distribution therefore cannot be completely ruled out. Dynamic geometric and photometric augmentation reduces direct memorisation of a fixed set of samples, but it is not a substitute for a larger and more diverse annotated mining dataset. For this reason, the robustness results should be interpreted within the scope of the available routes and the synthetic degradation protocol, rather than as definitive evidence of generalisation to all open-pit mining conditions.

## 6. Conclusions

The proposed model integrates drivable-area semantic segmentation and steering-direction classification within a single BiSeNetV1-based architecture, delivering a spatial estimate of the traversable surface and a lateral guidance signal under a hard parameter-sharing scheme. The methodology is well suited to the mining context for concrete practical reasons: road boundaries are loosely defined, dedicated datasets remain scarce, and producing semantic annotations is a slow manual process that does not scale to long video sequences.

The final evaluation focused on temporal testing over the complete Route A and Route B sequences. Under the selected Adaptive Clockwork configuration, the model achieved road-class IoU values of 94.92% and 94.70%, and Top-1 Accuracy values of 84.62% and 73.68% on Routes A and B, respectively. At inference time, Adaptive Clockwork reached GPU-only speeds of 165.19 and 168.01 FPS on Routes A and B, respectively, well past the frame-by-frame baseline. In the ADAS-type simulation, the method also raised the display-excluded end-to-end processing speed to approximately 37.51 FPS when the full perception pipeline, including visual postprocessing for the HMI overlay, was considered. The hardware benchmark further showed that the selected strategy can be executed across different GPU classes, providing lower-power edge-oriented references for the simulated ADAS perception pipeline.

Compared with Fixed Clockwork, DFF, and LLM, the proposed strategy stands out as a practical option for temporal video inference. Unlike DFF and LLM, Adaptive Clockwork requires neither an additional optical-flow module nor a trained scheduler nor temporal training with densely annotated sequences. The steering-direction classification output governs the keyframe update frequency autonomously, keeping dependence on long sequential annotations low while still enabling the model to operate on video at inference time. This adaptive behaviour was also evident under high semantic redundancy, where the simulated stop scenario produced accumulated GPU-only time savings whenever the visual scene changed slowly.

Overall, this work demonstrates that an efficient multitask perception module for mining ADAS can be built from a compact set of manually annotated images together with an adaptive temporal inference strategy. The results support classification-guided feature re-use as a viable approach to real-time perception on unstructured mining roads. Beyond the specific ADAS application, the methodology addresses a broader challenge in onboard vision sensing for unstructured environments: a data-efficient, computationally light framework that should extend naturally to other mobile sensing platforms operating under limited annotated imagery and tight real-time requirements.

## 7. Future Work

Several research directions remain open. They concern, broadly, extending the experimental validation of the system, optimising the ADAS pipeline, scaling the multitask formulation to new perceptual outputs, and evaluating the proposal from the perspectives of operator safety and real mining operations.

### 7.1. Robustness and Generalisation Under Extended Environmental Conditions

One pending direction is to reinforce the domain generalisation of the multitask model against severe variations of the open-pit mining environment, e.g., dense fog, suspended dust, rain, backlighting, lens-soiling degradation, and camera vibrations. The present study relied on data augmentation to improve robustness, but validation was limited to a restricted set of routes and conditions. A condition-oriented augmentation strategy that emulates plausible perceptual degradations from the available footage is therefore worth exploring. This direction is in line with recent work on domain generalisation and data augmentation for visual perception in transport [29], where synthetic diversity in visual conditions has been shown to improve robustness against domains not seen during training.

### 7.2. Multitask Approach Scaling and Multimodal Perception

The current model couples drivable-area semantic segmentation with steering-direction classification, but future versions could incorporate other tasks of clear operational value, e.g., obstacle detection and trajectory prediction. Multitask models carry well-known difficulties: task conflict, increased architectural complexity, higher data demand, additional computational load, reduced interpretability, and the practical challenge of scaling to new outputs. It is therefore worth examining explicit mitigation mechanisms, including dynamic loss balancing, uncertainty-based normalisation, and gradient-based optimisation methods that limit inter-task interference. Combining the camera with IMU, radar, LiDAR, GNSS, or other sensors should also improve robustness in scenarios where RGB vision degrades because of dust, fog, or low illumination.

### 7.3. ADAS Pipeline Optimisation

A direct improvement would be an asynchronous perception–HMI pipeline that decouples frame acquisition, preprocessing, GPU inference, postprocessing, visualisation and video/screen output via queues, double or triple buffering, and non-blocking CPU–GPU transfers. Such an architecture would let CPU-bound and GPU-bound stages overlap, easing bottlenecks and bringing the model’s measured acceleration closer to the real end-to-end throughput. Future evaluations should also target deployment on embedded hardware and report latency, energy consumption, memory usage, temporal stability, and execution robustness.

### 7.4. User Acceptance, Safety, and Ethics

In mining operations, an ADAS must do more than simply work: it has to be reliable, understandable, and accepted by the operators, particularly under fatigue, dust, and low visibility. Recent reviews of ADAS for safe and comfortable driving [16] examine how human–system interaction factors—perceived comfort, trust, and signal clarity—shape adoption in operational settings and offer a useful basis for designing user studies in the mining domain. Setting up a user-evaluation agenda with operators or drivers is therefore worthwhile: confidence, cognitive load, perceived utility, tolerance to false alarms, and HMI preferences are all candidates to be measured. On the impact and safety side, recent analyses of how ADAS affects road safety [8,71] provide a solid basis for defining operational KPIs and discussing risk in the mining context. From an ethical standpoint, a reasonable path forward is to formalise decision policies and system transparency, drawing on recent work on ethical decision algorithms for autonomous driving, both for unavoidable collision scenarios [72] and for obstacle-avoidance path planning under social and ethical constraints [73].

## Figures and Tables

**Table 1:** Temporal evaluation routes and their relationship with the training source. Route A has partial overlap with the training source, whereas Route B is an unseen route. In the trajectory graphs, orange denotes curved segments and light blue denotes straight segments.

Route Name	Training Source	Total Time (s)	Trajectory Graph
A	Partial overlap	103	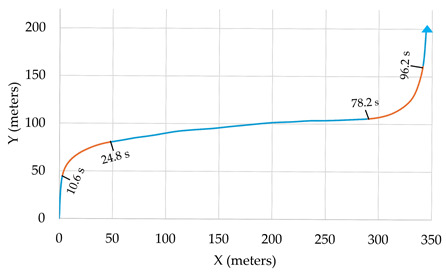
B	Unseen	56	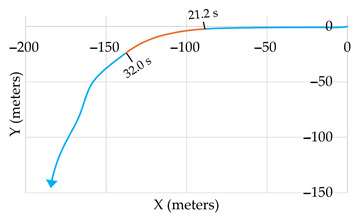

**Table 2:** Data augmentation parameters used during training of the multitask model.

Data Augmentation	Parameters
Rotate	Degrees between $\pm 5^{\circ }$
Flip	Horizontal
Photometric Distortion	Brightness delta = 32; Contrast = 0.5–1.5; Saturation = 0.5–1.5; Hue delta = 18
CLAHE	Clip limit = 3; Tile grid size = 7×7

**Table 3:** Main training hyperparameters of the proposed multitask model.

Parameter	Value
Batch size	4
Maximum iterations	40,000
Optimizer	AdamW
Initial learning rate	$1\times 10^{-3}$
Learning rate scheduler	Polynomial learning rate
Minimum learning rate	$1\times 10^{-5}$

**Table 4:** Validation performance and model statistics of the multitask BiSeNet and single-task BiSeNet variants. The evaluation was performed in frame-by-frame mode on the validation set used during training. BC and BS indicate the checkpoints selected according to best classification and segmentation performance, respectively.

Model	Checkpoint	Top-1 Acc (%)	mIoU (%)	FPS	Parameters (k)
Multitask	25,600 (BC)	96.25	97.33	60.41	85,190
	33,400 (BS)	96.25	97.64		
Classification Only	36,400	83.75	N/A	93.78	47,436
Segmentation Only	8200	N/A	98.72	69.62	85,187

**Table 5:** GPU-only and display-excluded end-to-end processing performance with keyframe statistics for the frame-by-frame reference and the fastest fixed-schedule configuration from each temporal strategy on Routes A and B. FPS values correspond to averages.

Parameter	BiSeNet FBF		F-CW (*k* = 100)		DFF (*k* = 100)	
	A	B	A	B	A	B
GPU FPS	55.54	55.33	184.30	184.30	86.98	86.62
E2E FPS	26.18	25.73	38.87	38.16	30.52	31.01
Keyframe	1031	561	11	6	11	6
Non-keyframe	0	0	1020	555	1020	555

**Table 6:** GPU-only and display-excluded end-to-end processing performance with keyframe statistics for the selected A-CW configuration and the fastest evaluated adaptive from temporal-inference methods on Routes A and B. FPS values correspond to averages.

Parameter	A-CW ($k_{c}$ = 10; $k_{s}$ = 100)		A-CW ($k_{c}$ = 50; $k_{s}$ = 100)		LLM (τ = 0.04)	
	A	B	A	B	A	B
GPU-Inference FPS	165.19	168.01	179.13	179.96	129.38	129.32
E2E Processing FPS	38.12	37.51	38.53	38.18	34.77	35.02
Keyframes	62	29	23	12	1	1
Non-keyframes	969	532	1008	549	1030	560

**Table 7:** Qualitative comparison of the evaluated temporal inference methods on Route A. For each method family, the configuration with the best classification inference performance was selected. The red overlay indicates the predicted drivable area, and the cyan arrow denotes the predicted steering direction.

Time (s)	DFF (*k* = 30)	LLM (τ = 0.01)	F-CW (*k* = 30)	A-CW ($k_{c}$ = 10; $k_{s}$ = 100)
10	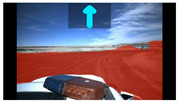	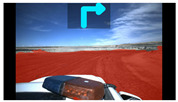	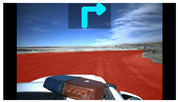	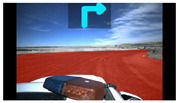
35	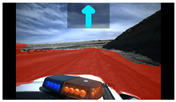	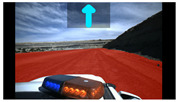	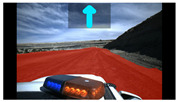	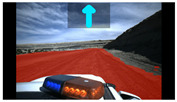
90	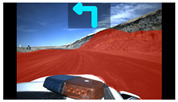	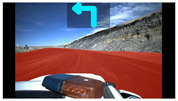	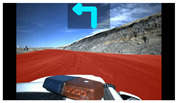	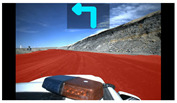

**Table 8:** Qualitative comparison of the evaluated temporal inference methods on Route B. For each method family, the configuration with the best classification inference performance was selected. The red overlay indicates the predicted drivable area, and the cyan arrow denotes the predicted steering direction.

Time (s)	DFF (*k* = 30)	LLM (τ = 0.01)	F-CW (*k* = 30)	A-CW ($k_{c}$ = 10; $k_{s}$ = 100)
15	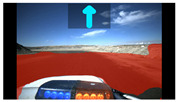	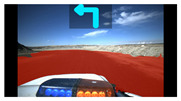	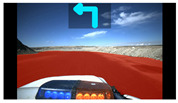	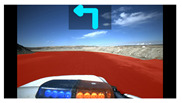
30	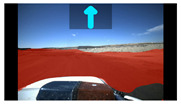	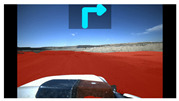	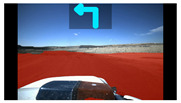	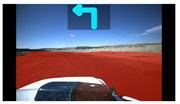
55	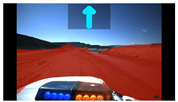	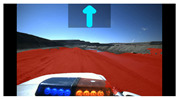	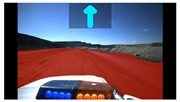	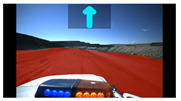

**Table 9:** Progressive ablation results of the selected Adaptive Clockwork strategy on Routes A and B. Bold values indicate the highest value obtained for each metric and route, while underlined values indicate the second-highest value.

Method	Cls.-Guided Selector	Context Reuse	Top-1 Acc (%)		mIoU Road (%)		GPU-Only FPS	
			A	B	A	B	A	B
MTL FBF *k*1	No	No	73.08	70.18	**95.93**	**96.57**	55.54	55.33
MTL F-CW *k*10	No	Yes	73.08	70.18	**95.93**	**96.57**	151.89	151.91
MTL F-CW *k*100	No	Yes	82.69	66.67	94.36	94.24	**184.30**	**184.30**
MTL A-CW *k*10 *k*100	Yes	Yes	**84.62**	**73.68**	94.92	94.70	165.19	168.01

**Table 10:** Computational complexity and GPU-only speed of the selected A-CW strategy and the video segmentation models.

Method	Parameters (M)	Route	GPU-Only FPS
Mask2Former	44.22	A	24.97
		B	25.06
MPVSS k30	84.39	A	154.20
		B	154.41
MPVSS k100		A	176.46
		B	176.86
A-CW ($k_{c}$ = 10, $k_{s}$ = 100)	85.19	A	165.18
		B	168.01

**Table 11:** Hardware benchmark of the selected A-CW strategy in the simulated ADAS pipeline. FPS values correspond to averages and E2E denotes display-excluded perception-pipeline throughput.

Hardware	GPU-Only Latency (ms)		E2E FPS		GPU-Only FPS		GPU Memory (MB)	
	A	B	A	B	A	B	A	B
RTX2000	22.52	22.45	24.86	24.54	44.41	44.55	2216	2226
L4	14.70	14.66	34.22	34.98	68.04	68.21	2295	2361
A100	5.63	5.91	47.53	47.92	168.90	169.16	2025	2025

**Table 12:** Robustness of the selected A-CW strategy under environmental visual degradations on Routes A and B. Luminosity and dust were evaluated at two severity levels. The table reports the degradation parameter, road-class IoU, and Top-1 Accuracy for each route.

Condition	Level	Parameter	mIoU Road (%)		Top-1 Acc (%)	
			A	B	A	B
Luminosity	Low	−2.5	26.55	35.84	72.34	80.70
	High	−1.5	87.33	90.92	78.72	61.40
Dust	Low	0.6	94.75	94.53	85.11	71.93
	High	1.0	94.61	94.01	86.17	73.68

**Table 13:** Robustness of the selected A-CW strategy under camera-related visual degradations on Routes A and B. Defocus blur and mud occlusion were evaluated at two severity levels. The table reports the degradation parameter, road-class IoU, and Top-1 Accuracy for each route.

Condition	Level	Parameter	mIoU Road (%)		Top-1 Acc (%)	
			A	B	A	B
Blur	Low	0.6	94.75	90.98	85.11	71.93
	High	1.0	94.61	81.39	86.17	73.68
Mud	Low	0.3	94.35	94.67	85.11	71.93
	High	0.6	94.22	94.65	85.11	73.68

## Data Availability

The complete research artefacts that support this study are publicly available through two complementary repositories, ensuring full reproducibility and FAIR compliance. All source code—the multitask BiSeNetV1 implementation, the four temporal-inference policies (Frame-by-Frame, Fixed Clockwork, Deep Feature Flow, Low-Latency Method, and the proposed Adaptive Clockwork), the training and evaluation pipelines, and the simulated HMI rendering module—is hosted on GitHub (https://github.com/ClaudioUrrea/mining-adas-acw, accessed on 9 June 2026). Simulation and evaluation data, including the 100 manually annotated binary masks, the IMU-derived per-frame metadata for Routes A and B, the train/validation/test splits, the trained model checkpoints (multitask Best-Classification and Best-Segmentation; single-task baselines; DFF and LLM baselines), the raw per-frame inference outputs for every evaluated configuration in CSV and Parquet, the statistical analyses, and the figure-source files that regenerate every figure of this paper, are deposited on Figshare (https://doi.org/10.6084/m9.figshare.32274630, accessed on 9 June 2026). All deposited artefacts include comprehensive metadata aligned with the FAIR (Findable, Accessible, Interoperable, Reusable) data-management principles, and the Figshare bundle ships a per-file SHA-256 manifest to verify integrity. Both repositories include documentation covering installation, a data dictionary, and step-by-step reproducibility instructions to support independent replication of the reported findings. The original AutoMine RGB frames and IMU streams used as source imagery are governed by the AutoMine project’s own license and are not redistributed in either repository; they can be obtained directly from the AutoMine authors [63]. The GitHub repository hosts the source code, training and evaluation scripts, configuration files, and documentation, while the Figshare repository hosts the derived annotations, trained checkpoints, raw per-frame results, statistical analyses, figure-source files, and the FAIR metadata.

## Abbreviations

The following abbreviations, listed in alphabetical order, are used in this manuscript:

ADAS	Advanced Driver Assistance System
A-CW	Adaptive Clockwork
BC	Best Classification Checkpoint
BiSeNet	Bilateral Segmentation Network
BS	Best Segmentation Checkpoint
CB	Convolution Block
CE	Cross-Entropy
CLAHE	Contrast Limited Adaptive Histogram Equalisation
Cls	Classification
CNN	Convolutional Neural Network
CPU	Central Processing Unit
DFF	Deep Feature Flow
DL	Deep Learning
DMS	Driver Monitoring System
DSS	Driver Support System
E2E	End-to-End
FBF	Frame-by-Frame
FC	Fully Connected Layer
F-CW	Fixed Clockwork
FFM	Feature Fusion Module
FPS	Frames per Second
GAP	Global Average Pooling
GPU	Graphics Processing Unit
HMI	Human–Machine Interface
IMU	Inertial Measurement Unit
IoU	Intersection over Union
LLM	Low-Latency Method
Mask2Former	Masked-attention Mask Transformer
mIoU	mean Intersection over Union
MTL	Multitask Learning
MPVSS	Mask Propagation for Efficient Video Semantic Segmentation
ONNX	Open Neural Network Exchange
RGB	Red–Green–Blue
ResNet	Residual Network
SS	Semantic Segmentation
ViT	Vision Transformer
VSS	Video Semantic Segmentation
YOLOP	You Only Look Once for Panoptic Driving Perception

## Appendix A

**Table A1:** Dataset traceability for training and temporal evaluation subsets.

Route	Purpose	Folder	Image ID Range
A	Test	oct00/right_cam 01/right_cam	1661922815–1661922918
B	Test	oct00/right_cam 04/right_cam	1662429010–1662429066
No name	Train	oct00/right_cam 01/right_cam	1661922791–1661923070
		oct00/right_cam 02/right_cam	1662253509–1662253589
			1662253877–1662253964
			1662254015–1662254070
			1662254231–1662254653

## Appendix B

**Table A2:** Adaptive Clockwork configurations evaluated using different keyframe update periods for curved and straight segments. Bold values indicate the highest value obtained for each metric and route.

Mode		Top-1 Acc (%)		mIoU (%)		mIoU Road (%)		FPS GPU-Only	
$k_{c}$	$k_{s}$	A	B	A	B	A	B	A	B
10	30	74.04	71.93	**96.81**	**96.52**	**95.67**	**95.44**	158.29	163.64
10	50	79.81	**73.68**	96.62	95.69	95.42	94.36	160.52	161.85
10	100	84.62	**73.68**	96.26	95.93	94.92	94.70	165.19	168.01
30	50	81.73	70.18	96.20	95.83	94.85	94.55	172.68	170.34
30	100	**85.58**	70.18	95.79	95.12	94.28	93.67	177.28	177.56
50	100	75.96	71.93	95.51	94.97	93.91	93.41	**179.13**	**179.96**

## Appendix C

**Table A3:** Main differences between the proposed Adaptive Clockwork approach and the sequence-based temporal baselines.

Aspect	A-CW	DFF Adaptation	LLM Adaptation
Training input	Isolated images	Video sequences with keyframe/non-keyframe structure	Pairs of frames
Temporal training	Not required	Required	Required in two stages
Context update policy	Determined by steering-direction classification	Determined by fixed scheduling	Determined by scheduler output and threshold τ
Propagation mechanism	Direct context feature reuse	Optical-flow-based feature propagation	Learned adaptive feature propagation
Additional temporal module	No additional trainable temporal module	FlowNetC-based motion module	Feature propagation module and adaptive keyframe selector
Model training initialization	BiSeNetV1 + ResNet-50 pretrained on ImageNet	BiSeNetV1 + ResNet-50 pretrained + FlowNetC pretrained on FlyingChairs dataset	Best-classification multitask BiSeNet checkpoint

## Appendix D

**Table A4:** Mapping between Deep Feature Flow and the proposed BiSeNet-based multitask DFF implementation.

Component	Purpose	Adaptation in BiSeNet Multitask
Feature Network ($N_{\mathrm{feat}}$)	Expensive feature extractor executed on sparse keyframes.	BiSeNet with ResNet-50
Task Network ($N_{\mathrm{task}}$)	Lightweight recognition network applied to the propagated feature map.	Implemented as the current-frame Spatial Path + Feature Fusion Module + task heads
Flow Function	Estimates optical flow between keyframe and current frame.	FlowNetC

## Appendix E

**Table A5:** Mapping between the Low-Latency Method and the proposed BiSeNet-based multitask LLM implementation.

Component	Purpose	Adaptation in BiSeNet Multitask
Lower Network ($S_{l}$)	Lightweight feature extractor executed on every frame.	BiSeNet Spatial Path
Higher Network ($S_{h}$)	Expensive semantic extractor executed mainly on keyframes.	BiSeNet Context Path
Low-Level Features ($F_{l}$)	Used to estimate temporal change and guide feature propagation.	Spatial features from keyframe/current frame
High-Level Features ($F_{h}$)	Semantic features reused from keyframes.	Context features from the last keyframe
AdapNet	Adapts/fuses low-level and propagated high-level features.	Covered by propagation refinement and Feature Fusion Module (FFM)
Feature Fusion	Combines current low-level features with propagated high-level features.	Feature Fusion Module (FFM)

## Appendix F

**Table A6:** GPU platforms used for the simulated ADAS hardware benchmark.

NVIDIA GPU Platform	Hardware Class	VRAM (GB)	Nominal Board Power (W)
NVIDIA RTX 2000 Ada	Compact workstation/edge-class GPU	16	70
NVIDIA L4	Edge/low-power data center GPU	24	72
NVIDIA A100 PCIe	Data center GPU	80	300

## Appendix G

**Table A7:** Confusion matrices for steering-direction classification on routes A and B using selected temporal inference methods. L, S, and R denote left, straight, and right classes, respectively. The rows correspond to routes A and B, while the columns correspond to the best-classification-performing configuration selected from each evaluated method family.

Route	F-CW *k* = 30	A-CW $k_{c}$ = 10, $k_{s}$ = 100	DFF *k* = 30	LLM τ = 0.01
A				
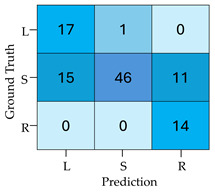
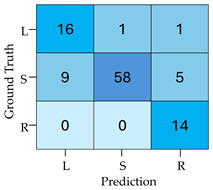
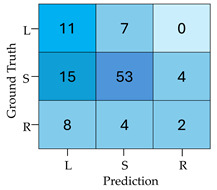
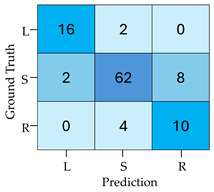
B				
